# Dopamine and Dopamine Receptors in Alzheimer's Disease: A Systematic Review and Network Meta-Analysis

**DOI:** 10.3389/fnagi.2019.00175

**Published:** 2019-07-11

**Authors:** Xiongfeng Pan, Atipatsa C. Kaminga, Shi Wu Wen, Xinyin Wu, Kwabena Acheampong, Aizhong Liu

**Affiliations:** ^1^Department of Epidemiology and Health Statistics, Xiangya School of Public Health, Central South University, Changsha, China; ^2^Department of Mathematics and Statistics, Mzuzu University, Mzuzu, Malawi; ^3^Department of Obstetrics and Gynaecology, University of Ottawa, Ottawa, ON, Canada; ^4^Ottawa Hospital Research Institute, Ottawa, ON, Canada; ^5^Department of Medicine & Therapeutics, The Chinese University of Hong Kong, Sha Tin, Hong Kong; ^6^Department of Public, School of Postgraduate Studies, Adventist University of Africa, Nairobi, Kenya

**Keywords:** dopamine, dopamine receptors, Alzheimer, systematic review, meta-analysis

## Abstract

**Background:** The dopaminergic system has been associated with the progression of Alzheimer's disease. But previous studies found inconsistent results regarding the relationship between Alzheimer's disease and dopamine when looking at dopamine receptor concentrations.

**Objective:** The aim of this review was to synthesize, using a random-effects model of meta-analysis, the link between the dopaminergic system and Alzheimer's disease.

**Methods:** A detailed analysis protocol was registered at the PROSPERO database prior to data extraction (CRD42018110798). Electronic databases of PubMed, Embase, Web of Science, and Psyc-ARTICLES were searched up to December 2018 for studies that examined dopamine and dopamine receptors in relation to Alzheimer's disease. Standardized mean differences (SMD) were calculated to assess group differences in the levels of dopaminergic neurometabolites.

**Results:** Seventeen studies met the eligibility criteria. Collectively, they included 512 patients and 500 healthy controls. There were significantly lower levels of dopamine in patients with Alzheimer's disease compared with controls (SMD = −1.56, 95% CI: −2.64 to −0.49). In addition, dopamine 1 receptor (SMD = −5.05, 95% CI: −6.14 to −3.97) and dopamine 2 receptor (SMD = −1.13, 95% CI: −1.52 to −0.74) levels were decreased in patients with Alzheimer's disease compared with controls. The results of network meta-analysis indicated that the rank of correlation with Alzheimer's disease from highest to lowest was dopamine (0.74), dopamine 2 receptor (0.49), dopamine 3 receptor (0.46), dopamine 4 receptor (0.33), dopamine 5 receptor (0.31), and dopamine 1 receptor (0.64).

**Conclusions:** Overall, decreased levels of dopaminergic neurotransmitters were linked with the pathophysiology of Alzheimer's disease. Nonetheless, there is a clear need for more prospective studies to validate these hypotheses.

## Introduction

Alzheimer's disease (AD) is a progressive neurodegenerative disorder with complex etiology (Scheltens et al., 2016). Early diagnosis of AD may help to implement timely interventions which could yield better prognosis and reduced burden of the disease (Cheng et al., 2015). Among the neurotransmitter abnormalities that have been investigated in AD, the dopaminergic system has been intensively studied as a key neurotransmitter system involved with emotion and cognition (Nardone et al., 2014). The dopaminergic system undergoes several changes during the neuropathological aging process. In general, some studies suggested that dopamine plays a major role in synaptic plasticity mechanisms (Hagena and Manahan-Vaughan, 2016). The progressive synaptic disarrangement, impairment of neurotransmission and cell loss would induce further the presence of extracellular deposits of amyloid protein, senile plaques, and intracellular fibrillary tangles, hence inducing symptoms of pre-dementia, like hypo-activity, gait disturbances and decline of cognitive functioning (Mattsson et al., 2017).

Moreover, experimental studies have demonstrated that the neurons forming the nigrostriatal pathway showed several pathologic changes, such as neurofilament triplet, neuropil threads, Aβ plaques, neuronal loss and decrease in dopamine content (Roostaei et al., 2017). Interestingly, in AD, the dysfunction of dopaminergic transmission has been hypothesized as a new player in the pathophysiology of AD (Nam et al., 2018). Dopamine acts through five different types of receptors, generally distinct in two main subclasses: D1-like [comprising the dopamine 1 receptor (D1R) and the dopamine 5 receptor (D5R)]; and D2-like [comprising the dopamine 2 receptor (D2R), dopamine 3 receptor (D3R) and the dopamine 4 receptor (D4R)] (Kumar and Patel, 2007).

Dopamine receptors are generally expressed in the limbic system and cortex, which is related to the control of mood and emotional stability. Furthermore, hippocampal D2R correlates with memory functioning in AD, and existing data suggested that dopamine acts through the D2-like receptors to increase cortical excitability and D1-like receptors to increase the release of cortical acetylcholine (Donthamsetti et al., 2018). However, existing data also suggested that dopamine levels were higher in AD patients than in controls, and dopamine receptors showed preferential marked increase of dopamine receptors in the hippocampus and cortex of the AD patients (Seeman et al., 1987; Sweet et al., 2001). Moreover, some dopaminergic system pharmacological treatments have been identified in AD, which suggests that the dopaminergic activity system may be a reasonable target for the pharmacological intervention of AD (Mitchell et al., 2011). However, other neurotransmitter systems are also associated with the process of dopaminergic system pharmacological treatments for AD, such as acetylcholine. For example, Martorana et al. (2009) found L-dopa administration, which modulates cholinergic cortical excitability, was able to improve the cognition ability of AD patients.

Overall, inconsistent results have been reported regarding the association of dopamine and dopamine receptor levels with AD (Seeman et al., 1987; Sweet et al., 2001; Kumar and Patel, 2007). Accordingly, it is necessary to further explore the role of dopamine in AD and which dopamine receptors have an effect on AD. A meta-analysis of the available related data would give some insight into the roles of dopamine and its five receptors in AD (Kumar and Patel, 2007). Thus far, no meta-analysis has been conducted and, therefore, the objective of this study was to conduct a comprehensive meta-analysis for the first time on the literature related to the relationship between dopamine and dopamine receptor concentration levels and AD, and quantify the strength of this relationship.

## Methods

### Search Strategy and Selection Criteria

This systematic review and meta-analysis has been registered, and the full protocol was uploaded to the International Prospective Register of Systematic Reviews website (CRD42018110798). In addition, it followed the Preferred Reporting Items for Systematic Reviews and Meta-Analyses (PRISMA) guidelines (Moher et al., 2009).

### Search Strategy and Selection Criteria

We searched relevant articles in four electronic databases: PubMed, Embase, Web of Science, and Psyc-ARTICLES. The search was restricted to all English articles published before December 2018. The search strategy was designed in consultation with experienced librarians, and it was structured as follows using keywords (search terms): (“alzheimers disease”:kw OR “alzheimer syndrome”:kw OR “alzheimer dementia”:kw OR “alzheimer”:kw OR “ad”:kw) AND (“dopamine”:kw OR “deoxyepinephrine”:kw OR “dopamine hydrochloride”:kw OR “hydrochloride, dopamine”:kw OR “intropin”:kw OR “receptors, dopamine”:kw OR “dopamine receptor”:kw OR “receptor, dopamine”:kw OR “dopamine receptors”:kw OR “receptors, dopamine d1”:kw OR “receptors, dopamine d2”:kw OR “receptors, dopamine d3”:kw OR “receptors, dopamine d4”:kw OR “receptors, dopamine d5”:kw). These search terms were adapted for the other databases whose detailed search strategies are shown in the supplemental material. Hand searching was conducted by XP and AC. Any inconsistencies between them were resolved by group discussion and consensus with a third party, AL.

### Eligibility Criteria

Studies were considered eligible if they (1) were case control studies, included AD cases and healthy controls; (2) described AD diagnostic criteria based on standardized criteria, such as that defined in the DSM or other international standardized criteria; and (3) reported mean and standard deviation (SD) of dopamine and dopamine receptor concentrations. Studies were excluded if they (1) were review articles or case reports; (2) studied AD in combination with other mental illnesses or in vascular dementia patients who used psychotropic medication or other medications which could influence the dopamine and dopamine receptor concentrations; (3) studied non-humans or were *in vitro* experiments; and (4) were gray literature (i.e., unpublished reports).

### Data Extraction

For the purpose of the meta-analysis, two independent investigators [XP and AC] extracted the following information according to the inclusion criteria specified above: (1) name of the first author and publication year; (2) country of the study; (3) study characteristics: mean age and standard deviation (mean, SD) of participants, gender distribution of participants, AD assessment method, and dopamine and dopamine receptors measurements comprising type of sample, sample bonder, storage temperatures (the meaning of frozen is whether samples were reported for frozen preservation in the study), and assay methods; and (4) mean and SD of dopamine and dopamine receptor concentrations. All the extracted data were organized in EpiData 3.0 and saved in Excel.

### Quality Evaluation

The Newcastle-Ottawa Quality Assessment Scale (NOS) was used to assess the quality of the eligible studies (Stang, 2010). Each eligible study was evaluated based on the three broad perspectives: (1) selection; (2) comparability; and (3) outcome. According to the pre-specified criteria of this scale, studies scoring 7–9, 3–6, and 0–3 points were graded, respectively, as high, moderate, and low quality.

### Statistical Analysis

In this study, meta-analyses were carried out using R software (version R i386 3.4.2). First, we performed meta-analysis of all enrolled studies to compare, one at a time, the concentrations of dopamine, D1R, D2R, D3R, D4R, and D5R between AD patients and healthy controls. This comparison was made using the standardized mean difference (SMD) of the foregoing concentrations between these two groups (Higgins et al., 2003). Precision of the SMD was described using corresponding 95% confidence intervals (CI). Heterogeneity between enrolled studies was quantified by the *I*^2^ statistic and assessed by the Cochran's Q-statistic (Pan et al., 2018a). *I*^2^ = 0% indicated no heterogeneity, and *I*^2^ = 100% indicated maximal heterogeneity. Second, the transitivity assumption was assessed visually to ensure that potential effect modifiers were balanced on average across comparisons (Pan et al., 2018b). If the assumption of transitivity was valid and the evidence formed a connected network, a meta-network analysis was conducted using a consistency model (White et al., 2012). The network meta-analysis or mixed comparison with a random-effects model within a Bayesian framework was performed using the GeMTC GUI (version 0.14.3) program (van Valkenhoef et al., 2012). All indirect comparisons were taken into account to arrive at an integrated effect of all included treatments based on all included studies. The first 20,000 iterations were discarded, and 50,000 further iterations were run. The model convergence was assessed by four Markov chains running simultaneously. In addition, the rank probabilities were calculated to obtain the hierarchy of each of the concentrations. The plots of rank probabilities were also established to compare the degree of influence by each of the preceding concentrations on AD. Third, sensitivity analysis involved redoing the meta-analysis by omitting each study in turn. Finally, in all analyses, the level of significance for the effect size estimation was set at 5%, and all tests were two-sided.

### Data Availability Statement

**Figure 1:**
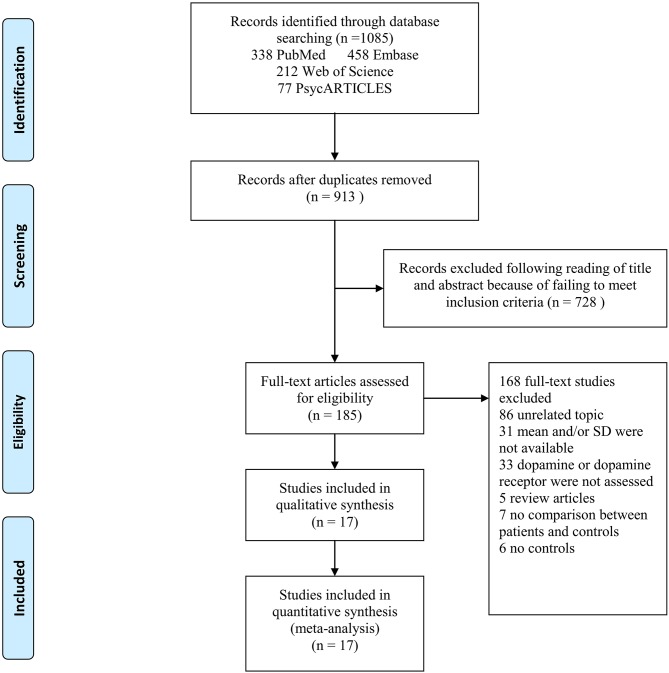
PRISMA flow chart of article selection, illustration of how eligible articles were selected. The process by which relevant studies retrieved from the databases were assessed and selected, or excluded. Preferred reporting items for systematic reviews and meta-analyses (PRISMA) diagram for study search.

**Figure 2:**
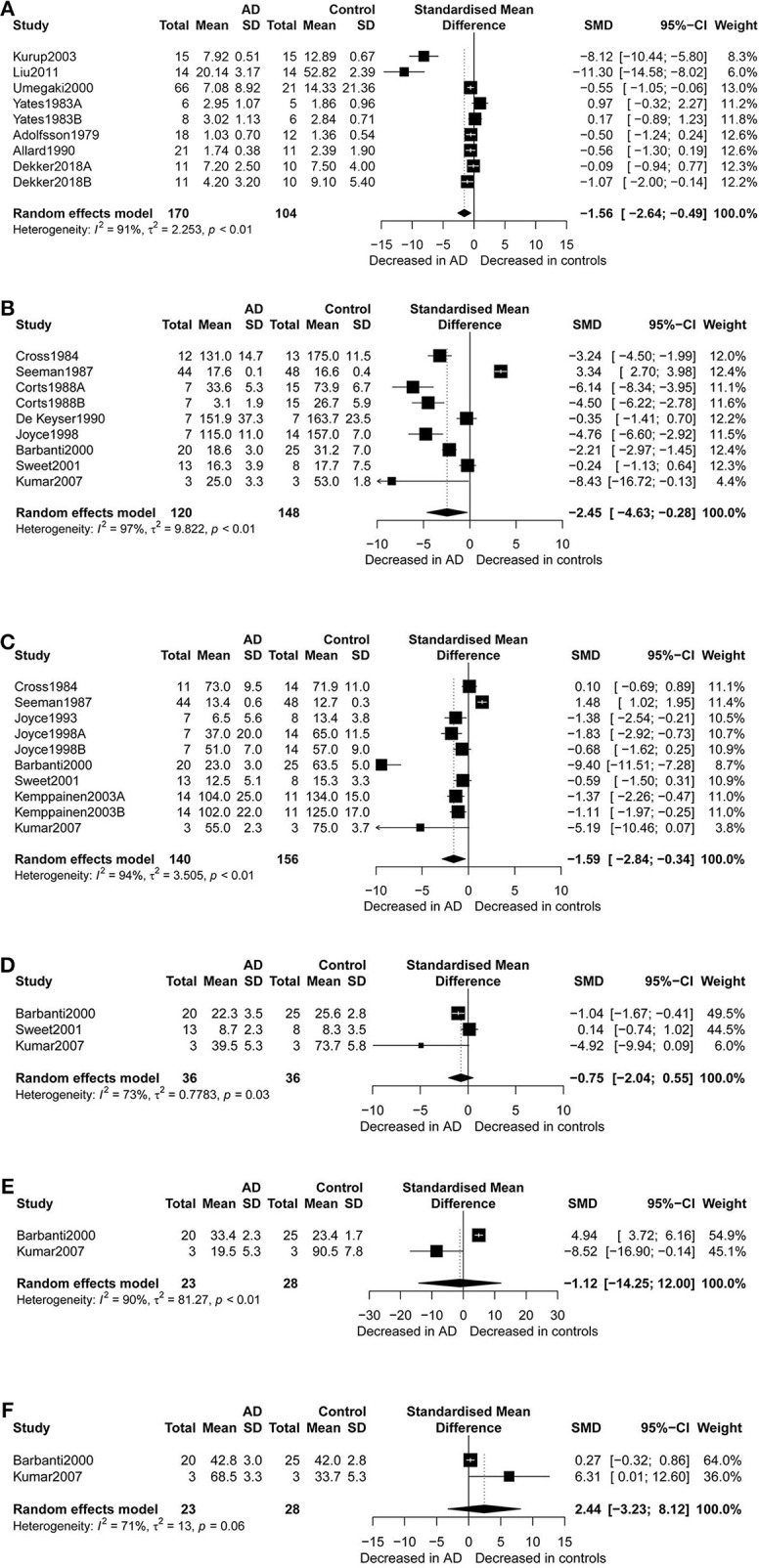
Forest plot of dopamine **(A)**, dopamine D1 receptors **(B)**, dopamine D2 receptors **(C)**, dopamine D3 receptors **(D)**, dopamine D4 receptors **(E)**, and dopamine D5 receptors **(F)** between AD participants and controls. Study effect sizes of dopamine and dopamine receptors and differences between AD and controls. Each data marker represents a study, and the size of the data marker is proportional to the total number of individuals in that study. The summary effect size for dopamine and dopamine receptors is denoted by a diamond. SMD, standardized mean difference; AD, Alzheimer's disease.

The data that support the findings of this study are available from PubMed, Embase, Web of Science, and PsycARTICLES, and shown in Figures 1, 2.

## Results

### Literature Search

The literature search yielded 1,085 relevant articles, of which 338 were from PubMed, 458 from Embase, 212 from Web of Science, and 77 from PsycARTICLES. After removing duplicates, 913 articles were retained. Following a review of the titles and abstracts of the 913 articles, 728 articles were excluded for failing to meet the inclusion criteria. Full review of the 185 articles resulted in further exclusion of 86 articles for being unrelated studies. Additionally, 33 articles were excluded for having no data on dopamine or dopamine receptor, 31 articles for not reporting means and SDs of dopamine or dopamine receptor, 7 articles for not comparing AD patients and controls, 5 articles for being review articles, and 6 articles for not reporting results for controls. A total of 17 articles met the eligibility criteria and were included in the final analysis (Figure 1).

### Characteristics of Eligible Studies

Table 1 presents the characteristics of the 17 eligible studies. These studies compared dopamine concentration levels between 170 AD patients and 104 healthy controls, and dopamine receptor concentration levels between 342 AD patients and 396 healthy controls. The NOS score of these studies varied between 5 and 8, with 9 studies of high quality and 8 of moderate quality.

Table 1Characteristics of studies included in meta-analysis of dopamine and dopamine receptors in Alzheimer's disease.

**Table:** 

**Study**	**N**	**Case**	**Control**	**Material**	**Country**	**NOS**	**Female**	**Mean age**	**AD assessment**	**Collection time**	**Methods**	**Frozen**
**A**												
**Dopamine**												
Kurup and Kurup, 2003	30	15	15	Plasma	India	5	0(0%)	67.5 ± 5.7	ICD-10	NR	HPLC	NR
Liu et al., 2011	28	14	14	Urine	China	5	0(0%)	81.7 ± 3.8	DSM-IV	NR	HPLC	−20°C
Umegaki et al., 2000	87	66	21	Plasma	Japan	7	66(100%)	82.5 ± 7.8	DSM-IV	AM 7	HPLC	−70°C
Yates et al., 1983	25	14	11	Brain	U.K	6	4(67%)	72.0 ± 18.0	Global dementia scale	NR	Other	−70°C
Adolfsson et al., 1979	30	18	12	Brain	USA	7	13(78%)	74.5 ± 7.2	Global dementia scale	NR	Other	−20°C
Allard et al., 1990	32	21	11	Brain	Sweden	8	0(0%)	82.0 ± 6.0	DSM-III	NR	HPLC	−70°C
Dekker et al., 2018	42	22	20	Brain	The Netherlands	7	8 (73%)	81.3 ± 7.6	DSM-IV	NR	HPLC	−80°C

**Table:** 

**B**											
**Dopamine receptors**									**Methods**	**Bonder**	
Barbanti et al., 2000	45	20	25	Peripheral blood lymphocytes	Italy	6	12(60%)	62.5 ± 3.7	Radioligand binding assay	[^3^H]7OH-DPAT/^[3^H]SCH 23390	NR
Cortés et al., 1988	44	14	30	Hippocampus/dentate gyrus	Switzerland	8	5(71%)	84.0 ± 2.0	Quantitative autoradiography	[^3^H]SCH 23390	−20°C
Cross et al., 1984	25	12	13	Putamen	U.K	6	7(58%)	79.0 ± 4.0	Quantitative autoradiography	[^3^H]Spiperone	−70°C
De Keyser et al., 1990	14	7	7	Frontal cortex	Belgium	5	3(43%)	60.0 ± 7.0	Quantitative autoradiography	[^3^H]SCH 23390	NR
Joyce et al., 1993	15	7	8	Hippocampus	USA	7	5(71%)	81.3 ± 6.1	Receptor autoradiography	[^125^I]epidepride	NR
Joyce et al., 1998	21	7	14	Caudate/nucleus accumbens	USA	7	4(44%)	76.0 ± 9.0	Receptor autoradiography	[^3^H]SCH 23390	−70°C
Kemppainen et al., 2003	50	28	22	Hippocampal/temporal lobes	USA	8	12(86%)	70.9 ± 6.6	Receptor autoradiography	[^11^C]FLB-457	NR
Kumar and Patel, 2007	6	3	3	Frontal cortex	USA	7	3(100%)	77.0 ± 3.1	Receptor autoradiography	[^3^H]SCH 23390	−80°C
Seeman et al., 1987	92	44	48	Striata	Canada	6	28(64%)	67.0 ± 1.3	Quantitative autoradiography	[^3^H]SCH 23390	NR
Sweet et al., 2001	21	13	8	Lewy bodies	USA	6	11(85%)	81.0 ± 10.0	Receptor autoradiography	[^3^H]SCH 23390	NR

*NR, not reported; N, total numbers of individuals; USA, United States of America; NOS, Newcastle-Ottawa Quality Assessment Scale; U.K, the United Kingdom; SCH 23390, [^3^H][R]-(+)-(–) chloro-2,3,4,5 tetrahydro-5-phenyl-1H-3-benzazepin-al-hemimaleate; 7OH-DPAT, [^3^H]7-hydroxy-N,N-di-n-propyl-2-aminotetraline; AD, Alzheimer's disease; DSM, Diagnostic, and Statistical Manual of Mental Disorders; HPLC, High performance liquid chromatography-tandem mass spectrometry; AM, Morning; NOS, Newcastle-Ottawa Quality Assessment Scale; ICD, International Classification of Diseases*.

### Overall Comparison

Figure 2 presents the results of random-effects meta-analysis. For the 9 studies which compared dopamine concentration levels between AD patients and healthy controls, significantly lower concentration levels of dopamine were observed in patients with AD compared with controls (*k* = 9, SMD = −1.56, 95% CI: −2.64 to −0.49), and heterogeneity was considerable (*I*^2^ = 91.30%, Figure 2A). A similar analysis of 9 studies which compared D1R concentration levels between AD patients and healthy controls showed that D1R concentration levels were significantly lower in AD patients as compared to controls (*k* = 9, SMD = −2.45,95% CI: −4.63 to −0.28, Z = −2.21, *p* = 0.027), and with high heterogeneity (*I*^2^ = 96.70%, Figure 2B). We also found that D2R concentration levels were significantly lower in patients with AD compared with controls (SMD = −1.59; 95% CI, −2.84 to −0.34; *I*^2^ = 94.10%; *p* = 0.013) (Figure 2C). There was no significant difference in the concentration levels of D3R, D4R, and D5R between the two groups.

### Subgroup Analyses

Table 2 shows the results of subgroup analyses. Lower dopamine concentration levels were observed in AD patients than in healthy controls (SMD = −1.59, 95% CI: −2.88 to −0.30) for studies with patients aged 80 years or older. There were no identified differences in dopamine concentration levels between the two groups of participants for studies with AD patients <80 years old. Furthermore, dopamine concentration levels were significantly lower in AD patients than in healthy controls (SMD = −2.27, 95%CI: −4.36 to −1.18) when high performance liquid chromatography-tandem mass spectrometry (HPLC) was used in the assay of dopamine.

**Table 2:** Subgroup Analysis of dopamine, dopamine 1 receptor, dopamine 2 receptor between AD participants and controls.

	**Number of studies**	**SMD (95% CI)**	**Z**	***P*-value**	**Heterogeneity**		
					**Q statistic (DF; p value)**	**τ^2^**	**I^2^**
**DA**							
All	9	−1.56 [−2.64; −0.49]	−2.84	0.004	91.61 8 <0.0001	2.25	91.30%
**MATERIAL**							
Brain	6	−0.33 [−0.69; 0.03]	−1.80	0.072	8.11 5 0.1504	0.13	38.30%
Other	3	−6.53 [−13.53; 0.47]	−1.83	0.067	77.06 2 <0.0001	36.86	97.40%
**MEAN AGE**							
>80	5	−1.59 [−2.88; −0.30]	−2.41	0.016	43.21 4 <0.0001	1.77	90.70%
≤ 80	4	−1.61 [−3.98; 0.76]	−1.33	0.183	48.07 3 <0.0001	5.32	93.80%
**GENDER**							
Female	6	−0.38 [−0.70; −0.06]	−2.35	0.018	8.43 5 0.1342	0.12	40.70%
Male	3	−6.53 [−13.47; 0.40]	−1.85	0.065	71.53 2 <0.0001	36.18	97.20%
**ASSAYED METHODS**							
HPLC	6	−2.77 [−4.36; −1.18]	−3.41	0.001	82.06 5 <0.0001	3.34	93.90%
Other	3	0.08 [−0.74; 0.91]	0.20	0.840	4.00 2 0.1354	0.27	50.00%
**D1R**							
All	9	−2.45 [−4.63; −0.28]	−2.21	0.027	245.82 8 <0.0001	9.82	96.70%
**COUNTRY**							
USA	3	−3.39 [−7.55; 0.77]	−1.60	0.11	21.83 2 <0.0001	10.21	90.80%
Other	6	−2.11 [−4.95; 0.73]	−1.45	0.146	218.94 5 <0.0001	12.11	97.70%
**MEAN AGE**							
≤ 80	6	−3.93 [−6.13; −1.72]	−3.49	0.001	49.81 5 <0.000	5.99	90.00%
>80	3	0.26 [−3.39; 3.91]	0.14	0.887	125.46 2 <0.0001	10.22	98.40%
**FROZEN**							
Yes	4	−5.05 [−6.14; −3.97]	−9.17	<0.0001	2.07 3 0.5570	0.00	0.00%
No	5	−0.52 [−3.00; 1.96]	−0.41	0.68	161.93 4 <0.0001	7.77	97.50%
**D2R**							
All	10	−1.59 [−2.84; −0.34]	−2.49	0.013	152.23 9 <0.0001	3.51	94.10%
**COUNTRY**							
USA	7	−1.13 [−1.52; −0.74]	−5.70	<0.0001	6.51 6 0.3690	0.02	7.80%
Other	3	−2.37 [−5.91; 1.18]	−1.31	0.191	100.52 2 <0.0001	9.39	98.00%
**NOS**							
High	6	−1.26 [−1.69; −0.82]	−5.70	<0.0001	4.83 5 0.4370	0.00	0.00%
Other	4	−1.83 [−4.36; 0.69]	−1.42	0.154	107.85 3 <0.0001	6.26	97.20%
**GENDER**							
Female	4	−1.07 [−1.58; −0.56]	−4.13	<0.0001	3.86 3 0.2775	0.09	22.20%
Male	6	−1.74 [−3.64; 0.16]	−1.80	0.072	132.07 5 <0.0001	5.28	96.20%
**METHODS**							
Receptor autoradiography	7	−1.13 [−1.52; −0.74]	−5.70	<0.0001	6.51 6 0.3690	0.02	7.80%
Other	3	−2.37 [−5.91; 1.18]	−1.31	0.191	100.52 2 <0.0001	9.39	98.00%
**BONDER**							
[3H]SCH 23390	6	−2.29 [−4.50; −0.09]	−2.04	0.041	132.00 5 <0.0001	6.60	96.20%
Other	4	−0.89 [−1.64; −0.15]	−2.35	0.019	8.00 3 0.0460	0.36	62.50%
**FROZEN**							
Yes	4	−0.95 [−2.11; 0.21]	−1.61	0.108	10.78 3 0.0130	0.86	72.20%
No	6	−1.86 [−3.75; 0.03]	−1.93	0.054	137.53 5 <0.0001	5.24	96.40%

*DA, dopamine; AD, Alzheimer's disease; D1R, dopamine 1 receptor; D2R, dopamine 2 receptor; the meaning of frozen is whether samples were reported for frozen preservation in the study; NR, not reported; USA, United States of America; DSM, Diagnostic, and Statistical Manual of Mental Disorders; HPLC, High performance liquid chromatography-tandem mass spectrometry; AM, Morning; NOS, Newcastle-Ottawa Quality Assessment Scale; U.K, the United Kingdom; ICD, International Classification of Diseases; SCH 23390, [^3^H][R]-(+)-(–) chloro-2,3,4,5 tetrahydro-5-phenyl-1H-3-benzazepin-al-hemimaleate; SMD, standardized mean difference; DF, degrees of freedom*.

Subgroup analyses for D1R found that D1R concentration levels were lower in patients with AD compared with controls (SMD = −5.05, 95% CI: −6.14 to −3.97) when the samples were cryopreserved. This result was with no heterogeneity (I^2^ = 0%). On the other hand, there were no group differences for samples which were preserved at room temperature.

Additionally, D2R concentration levels were significantly lower in AD patients than in healthy controls (SMD = −1.13, 95%CI: −1.52 to −0.74), with low heterogeneity (I^2^ = 7.80%), when USA studies were considered. Apparently, subgroup analysis of studies with high NOS scores indicated notably lower heterogeneity (I^2^ = 0%) than when meta-analysis was performed for all the studies together. Also, in the subgroup analysis by gender, it was observed that females with AD presented with lower concentration levels of D2R than female controls (SMD = −1.07, 95%CI: −1.58 to −0.56), but no such significant difference was apparent between males. Finally, significantly lower D2R concentration levels were also observed in AD patients compared to the controls when receptor autoradiography was used in the assay of D2R.

### Relative Ranking of Six Groups

**Figure 3:**
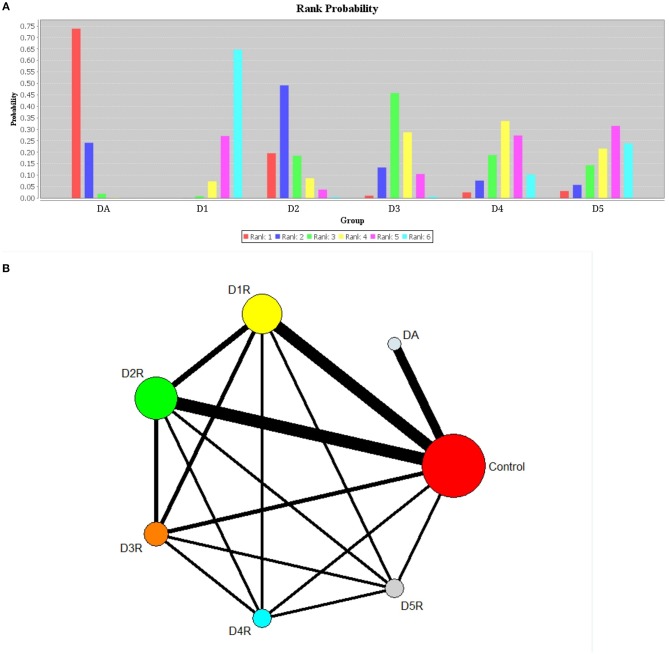
Plots of rank probabilities for six groups of dopaminergic **(A)** and network diagram representing direct comparisons among classes **(B)**. DA, dopamine; D1, dopamine 1 receptor; D2, dopamine 2 receptor; D3, dopamine 3 receptor; D4, dopamine 4 receptor; D5, dopamine 5 receptor. **(A)** Six groups of different ranks are represented by different color bands, and the highest rank coefficient in each group is the order ranking of this group. **(B)** The width of lines represents the number of studies in which each direct comparison is made. The size of each circle represents the number of people who received each study.

Based on the results of rank probabilities as shown in Figure 3A, we could easily find the ranking of each of the following concentrations (ordered from highest priority to lowest): dopamine, D2R, D3R, D4R, D5R, and D1R. A higher probability of achieving rank 1 indicated a higher influence on AD. As indicated by the results, dopamine ranked the highest in rank score, which means it had a strong correlation with AD. Moreover, according to the rank probability charts, the rank of correlations with AD, arranged from highest to lowest, are given as follows: dopamine (0.74), D2R (0.49), D3R (0.46), D4R (0.33), D5R (0.31), D1R (0.64). Figure 3B shows the network of comparisons among classes, and we have uploaded the table of the rank probabilities as the Appendix in Supplementary Material. The width of lines represents the number of studies in which each direct comparison was made. The size of each circle represents the number of people who participated in each study.

### Sensitivity and Bias Analysis

Sensitivity analysis indicated that any single study or a cluster of studies sharing some characteristics influenced little change in SMD and its corresponding 95% CI. Publication bias was not reported for dopamine and dopamine receptors because the number of studies reporting dopamine was <10 for each comparison.

## Discussion

### Main Findings

To the best of our knowledge, this is the first systematic review and meta-analysis to explore the relationship between AD status and dopamine, and AD status and dopamine receptors. To date, a number of factors have been proposed to cause damage to the brain, including the presence of extracellular deposits of amyloid protein, senile plaques, and intracellular fibrillary tangles. All these factors are responsible for progressive synaptic disarrangement, cell loss and impairment of neurotransmission. Also, results of animal and clinical studies support the hypothesis that disruption of the dopaminergic system is associated with the pathophysiology of AD (Nam et al., 2018).

More recently, it has been proven by some studies of genetic epidemiology that polymorphism of dopamine beta-hydroxylase (DBH) may be associated with the process of AD pathophysiology progression (Belbin et al., 2019). However, it is not clear that it is shown if the relative dopaminergic deficit in AD patients is the cause (partially genetic) or the effect of the disease, since dopaminegic loss occurs with age (Bäckman and Farde, 2001; Li et al., 2010).

The hypothesis of an association between AD and the dopaminergic system is not only supported by genetic studies, but also experimental and clinical studies. For instance, it is widely accepted that dopamine, which derives from the ventral tegmental area (VTA) dopaminergic neurons, modulates and projects mainly cerebral cortex, nucleus accumbens and hippocampal functions, including control of voluntary movement, memory and synaptic plasticity (Nobili et al., 2017). It has also been demonstrated that the overall dopaminergic system (dopamine and dopamine receptors) may decline with age (Karrer et al., 2017). Moreover, in a recent research, Nam et al. provided experimental evidence showing that dopamine and its structural analogs can reduce oxidative stress and inflammation, which are triggered by Aβ through the diminishing of the induction of inflammatory mediators at the neurofibrillary tangle formation and early “pre-plaque” stage during the progression of AD (Nam et al., 2018). Although no cell death occurs in early “pre-plaque” stage in the hippocampus, dopaminergic neuron loss and dopaminergic degeneration could be detected in VTA.

Also, in another recent study, Dekker et al. provided clinical evidence showing that dopamine and its derivatives, such as homovanillic acid and 3,4-dihydroxyphenylacetic acid, are lower in down syndrome with early-onset AD (Dekker et al., 2018). Additionally, Koch et al. demonstrated that dopamine D2/D3 agonists such as rotigotine may rescue the AD patients by restoring the cortical plasticity, which may suggest new strategies of therapeutics in AD (Koch et al., 2014). In line with the preceding evidence and hypothesis, our findings suggested that dopamine concentration levels were significantly lower in AD patients than in controls. This is consistent with the results of the localization studies of dopamine receptors, which showed a preferential marked decrease of D2-like receptors in the hippocampus and frontal cortex of the brains of AD patients, whose density progressively declined with aging (Tiernan et al., 2018). Moreover, several lines of investigation have shown that by acting through the D2-like receptors, dopamine increases cortical excitability, whereas via D1-like receptors dopamine increases cortical acetylcholine release. These observations support the idea that disruption of the dopaminergic system is associated with the pathophysiology of AD, and modulation of the dopaminergic system may lead to a novel therapeutic approach to AD.

Interestingly, the clinical studies have shown that rotigotine, a dopamine D2 agonist, induces changes in both cortical excitability (increased) and central cholinergic transmission (restored) in AD patients (Martorana et al., 2013). Thus, in AD, the dysfunction of dopaminergic transmission has been hypothesized as a new player in the pathophysiology of AD. Overall, these results support hypo-dopaminergic function in AD and are in line with our finding of decreased concentration levels of dopamine and dopamine receptors in patients with AD.

### Subgroup Analyses

As regards dopamine, subgroup analyses indicated that age of subjects and assay method of dopamine significantly explained heterogeneity. In particular, decreased dopamine concentration levels were observed in patients aged at least 80 years. Thus far, a number of factors have been proposed to cause age-induced damage to the brain, including oxidative stress, free radical damage, and intracellular fibrillary tangles, which can be modified by aging, and have been associated with occurrence of AD (Pohanka, 2018). Although the underlying mechanisms of this selective impairment remain poorly understood, animal and human data suggest that dopaminergic modulation may be particularly relevant for damage to the brain. Specifically, some age-related changes in the expression of genes are associated with neurotrophic factor signaling and the regulation of tyrosine hydroxylase activity (Borba et al., 2016). Therefore, future clinical trials are needed to verify the potential therapeutic effectiveness of dopaminergic drugs in AD patients, especially the patients aged 80 years or older.

As regards the assay method used, we observed no significant difference in the concentration levels of dopamine between AD patients and healthy controls when the assay method was not HPLC. It may suggest that compared with other technologies for detecting dopamine, HPLC may be more reliable. Considering the assay method used, it was observed that there was no significant difference in the concentration levels of dopamine between AD patients and healthy controls when assay method was not HPLC. Therefore, it may be suggested that compared with other technologies for detecting dopamine, HPLC could be more reliable. Noteworthily, there are variations in the methodologies used for the different measurements made over almost four decades ago (1979 to 2018 for dopamine quantification, and 1984 to 2007 for dopamine receptors). For example, Barnum described a spectrophotometric method for determining dopamine, catechol, epinephrine and other aromatic vicdiols in 1977 (Barnum, 1977). This method is simple to operate, but with low sensitivity. Additionally, Dayton et al. (1979) reported a method for evaluating the dopamine and 5-hydroxytryptamine in synaptosomes by electrochemical measurement. However, in some biological samples, the oxidative peak potential of coexisting substances such as epinephrine was similar to that of dopamine, so the determination of dopamine was seriously disturbed (Wagner et al., 1979).

Furthermore, Westerink and Van Oene (1980) reported a highly sensitive method for the determination of dopamine metabolism in the mice, based on isolation on Sephadex columns in combination with HPLC. This method is accurate, suitable, rapid, and simple, and is also currently widely used in the quantification of dopamine. Therefore, most of the studies involved in this systematic review and network meta-analysis used the HPLC method to detect dopamine. Later, Nozaki et al. (1996) developed and validated a new method for dopamine using chemiluminescence. This method attracted more and more attention because of its high sensitivity, simplicity and rapidity (Saqib et al., 2019). Additionally, Li et al. (2000) reported a semi-automated method detection of L-dopa and dopamine in plasma by electrospray LC/MS. This method has high detection sensitivity, but it is complex to operate and requires a high level of experimental equipment, so it is difficult to be widely used. Moreover, Shinohara and Wang (2007) developed and validated a new enzyme-catalyzed luminescence method for real-time detection of dopamine release from a nerve cell.

Also, concerning dopamine receptors, Stafanini and Clement-Cormier (1981) reported a new imaging of the [^3^H]Spiroperidol method in 1981 for the determination of D-2 dopamine receptors by single-photon emission computed tomography (SPECT). In addition Ehrin et al. (1985) found a new potent dopamine receptor antagonist to determine cerebral dopamine receptors by visualizing positron emission tomography (PET). Both SPECT and PET have been the mainstream measurement methods for over 30 years to assess dopamine receptor quantification (Booth et al., 2015), and most of the studies that provided data for this systematic review and network meta-analysis used these methods. However, there are some new methods that are worthy of attention. For example, Xiao and Bergson (2013) reported a new method for detecting and quantifying levels of dopamine receptors by using protein biotinylation and enzyme-linked immunoabsorbent assay (ELISA). Also, Navarro et al. (2013) reported sequential bioluminescence resonance energy transfer (BRET) and fluorescence resonance energy transfer (FRET) technologies for detecting dopamine receptors. These new methods might be promising tools for investigating *in vivo* the role of dopamine receptors in AD.

Furthermore, subgroup analyses found, with no heterogeneity, that the D1R concentration levels were lower in patients with AD compared with controls when the samples were cryopreserved. These results suggest that cryopreservation is conducive for a sample to retain high viability with protein, and physiological signatures consistent with D1R.

Also, subgroup analysis indicated that the concentration levels of D2R were significantly lower in AD patients than in healthy controls with low heterogeneity when USA studies were considered. This result may suggest that different countries or regions may form different homogenous groups when investigating the relationship between dopamine and AD. However, since more studies for this systematic review and meta-analysis were conducted in the United States, more future investigations are needed in other countries to enable verification of this hypothesis. We could have evaluated heterogeneity in terms of social political economic, ethnicity, technical level and cultural factors but these characteristics were rarely reported in the eligible studies (Lopez-Bastida et al., 2006). Thus, we considered country of study as a likely substitute because it contains all those characteristics.

In the subgroup analysis by gender, it was found that females with AD had lower concentration levels of D2R than female controls, and no such significant difference was apparent between males. This is consistent with some evidence which showed that women are at higher risk than men of developing AD (Ferretti et al., 2018). Moreover, other research found that multimodality brain imaging indicated gender differences in the development of the AD endophenotype, with postmenopausal women and perimenopausal women exhibiting increased indicators of the AD endophenotype than men, suggesting that the preclinical AD phase presents early in the female aging process and coincides with the endocrine transition of perimenopause. In addition, current studies of individuals with AD provide evidence of alterations in the neuroendocrine system that dopamine and acetylcholine are affected by gender steroid hormones (Giacobini and Pepeu, 2018). One explanation could be that gender hormones exert trophic effects on the cholinergic system, while acetylcholine is involved in dopaminergic mediators which are responsible for progressive synaptic disarrangement, impairment of neurotransmission and cell loss. Gender-related differences in neural anatomy and function are starting to emerge, and gender might constitute an important factor for AD patient stratification and personalized treatment. Finally, we observed significantly lower D2R concentration levels in AD patients compared to the controls when receptor autoradiography was used in the assay of D2R. This result suggests that compared with the other technologies for detecting D2R, receptor autoradiography may be more reliable.

Moreover, previous studies have shown that some dopaminergic system pharmacological treatments have been identified in AD, suggesting that the dopaminergic activity system may be a reasonable target for the pharmacological intervention of AD. As shown in Table 3, a clinical trial revealed that rotigotine, a dopamine agonist, may restore altered cortical plasticity in AD (Koch et al., 2014). Another prospective clinical trial showed that treatment of a dopamine receptor antagonist, risperidone, in more severe dementia was showing significant therapeutic effect, but not significant in other subgroups (Mintzer et al., 2006). For dopamine precursor, a case-control study showed that levodopa administration was able to improve the cognition ability of AD patients (Martorana et al., 2009). For dextroamphetamine, a dopamine uptake inhibitor, a prospective clinical trial showed that it could improve apathy in AD patients (Lanctôt et al., 2008). Devanand et al. (2011) also found haloperidol, a D2 receptor antagonist, which was able to restore the Clinical Global Impression-Change score in AD in a 6-month, randomized, double-blind, placebo-controlled pilot discontinuation trial. Interestingly, amantadine is another drug with dopaminergic effects that has been demonstrated to delay dopamine reuptake and stimulate the release of dopamine. Drayton et al. investigated amantadine in the treatment of apathy and found patients (56.7%) were rated as “much improved” or better on the clinical global impression scale (Drayton et al., 2004). Methylphenidate is a central nervous system psychostimulant that increasing the synaptic dopamine levels. A randomized, placebo-controlled crossover, double-blind trial found greater improvement in symptoms of apathy in AD patients taking Methylphenidate than placebo (Herrmann et al., 2008).

**Table 3:** Characteristics and findings of dopaminergic system related pharmacological treatments on AD.

**Study**	**ClinicalTrials.gov Identifier**	**Participant**	**Intervention**	**Finding**
Koch et al., 2014	NCT03250741	Thirty AD patients and 10 healthy subjects	Dopamine agonist rotigotine (RTG)	Dopamine agonists may restore the altered of cortical plasticity in AD
Cummings, 2015	NCT02359552	Fifty AD patients	Twenty-five receive Rasagiline and 25 receive placebo	Unpublished
Bennett and Burns, 2011	NCT01388478	Twenty AD patients	R(+)-pramipexole will be taken as a liquid 16 weeks	Unpublished
Jessen, 2011	NCT01047254	One hundred and ten AD patients	A 12-week of Bupropion(dopamine uptake inhibitor)for AD	Unpublished
Mintzer et al., 2006	NCT00034762	Four hundred seventy-three AD patients	Placebo (*N* = 238) or 1.0 to 1.5 mg risperidone (dopamine receptor antagonist) per day (*N* = 235)	Treatment by risperidone in more severe dementia showed significant differences but not for other subgroups.
Martorana et al., 2009	NR	Ten AD patients	Single oral dose of levodopa (dopamine precursor)	Levodopa administration was able to improve cognition ability of AD patients
Lanctôt et al., 2008	NCT00254033	Twenty AD patients	Patients were given a single 10 mg dose of Dextroamphetamine(dopamine uptake inhibitor)	AD patients were responsive to the rewarding effects of dextroamphetamine
Devanand et al., 2011	NCT00009217	Twenty-two AD patients	Twenty weeks of haloperidol (Haloperidol D2 receptor antagonist)	Haloperidol open treatment was efficacious
Drayton et al., 2004	NR	Thirty AD patients	Amantadine for AD patients	Patients (56.7%) were rated as “much improved” or better on the clinical global impression scale
Herrmann et al., 2008	NR	Thirteen AD patients	Methylphenidate(20 mg/day) or placebo	Greater improvement in symptoms of apathy in patients taking Methylphenidate than placebo

*DA, dopamine; AD, Alzheimer's disease; D2R, dopamine 2 receptor; NR, not report*.

Recently, G protein coupled receptor (GPCR) homodimer and G protein provides a main functional unit and has emerged as potential novel targets for drug development (Casadó-Anguera et al., 2016). Moreover, dopamine is distributed in CNS and its physiological effects are mediated by five closely related GPCRs that are divided into two major subclasses: the D1-like and the D2-like receptors (Franco et al., 2007; Cortés et al., 2016). On the basis of this interaction, some studies of heterotetrameric structures have been proposed for D1R-D3R and adenosine A2Areceptor (A2AR)-D2R heteromers (Cortés et al., 2016). It is widely known that A2AR antagonists have been proposed as potential drugs for the treatment of Parkinson's disease (Cavić et al., 2011). Interestingly, existing research has also decoded the signaling of a GPCR heteromeric complex, which may reveal therapeutic strategies for disorders including Parkinson, schizophrenia and dementia (Fribourg et al., 2011).

### Relative Ranking of Six Groups

Relatively, network meta-analysis indicated that the rank of correlation between the six treatments and AD was in the following descending order of the strength of the correlation: dopamine, D2R, D3R, D4R, D5R, and D1R. These results are consistent with previous studies on dopaminergic system in patients with AD. Moreover, it is known that by acting through the D2-like receptors, dopamine increases cortical excitability, while via D1-like receptors it increases cortical acetylcholine release. This result may imply that D2-like receptors play a more important role in the development of AD than D1-like receptors (Hagena and Manahan-Vaughan, 2016).

This hypothesis has been supported by recent research, since medications intended to treat acetylcholine deficiency have not been very effective for AD. In addition, these findings are corroborated by clinical studies, suggesting that stimulation of primary motor cortex by TMS in AD patients revealed an impairment of central cholinergic activity that can be transiently restored by the administration of Levodopa or rotigotine, or a dopamine D2-like agonist (Martorana et al., 2013). Also, this was supported by other clinical studies which indicated that alteration of cortical plasticity is rescued in AD patients who are treated with rotigotine, thus testifying that dopaminergic stimulation might reveal a therapeutic strategy for AD. Although the molecular underpinnings of brain dopaminergic system degeneration in AD remain to be defined, the foregoing results might open up new perspectives in early diagnosis and provide novel targets for pharmacological intervention.

## Study Limitations

First, each eligible study for this meta-analysis had a small sample size, perhaps due to the need for advanced technology costs in order to perform such studies successfully. The small sample sizes of the eligible studies might have compromised the significance of the conclusions (Sweet et al., 2001). In this regard, it is necessary to further explore which dopamine receptors may have an effect on AD, hence more multiple-center, large-sample and high-quality randomized controlled trials are needed. Second, given the high heterogeneity among the eligible studies, the comparability among studies was limited. Meanwhile, different sample sources and degree of the AD may lead to significant heterogeneity. In addition, different sample sources will lead to different application directions in the future. The data of brain tissue and PET in the brain can provide suggestions for targeted drugs, while the data of plasma or urine can provide a reference for biomarkers. Although some subgroup analyses explained the source of heterogeneity, some factors that may influence dopamine and dopamine receptors concentration levels, such as BMI (body-mass index), smoking cigarettes, drinking, physical activity, and blood pressure, were neither measured nor adjusted in the original eligible studies. Finally, the literature used for this meta-analysis originated from cross-sectional studies, which could not make any causality inference.

## Conclusions

Our meta-analysis has shown that there is evidence to suggest that dopamine, D1R and D2R concentration levels were decreased in patients with AD compared with controls. In addition, decreased levels of dopamine and D2-like receptors were linked with the pathophysiology of AD because of their strong higher rank correlations with AD. Nonetheless, there is a clear need for more prospective studies to validate these hypotheses.

## Author Contributions

XP and AL contributed to the study design, while XP and AK contributed to the data collection. Statistical analyses and interpretation of results were performed by XP and AK, while XP, XW, KA, AL, and SW drafted the manuscript and edited the language. All the authors participated in the critical revision and approved the final version of the manuscript.

### Conflict of Interest Statement

The authors declare that the research was conducted in the absence of any commercial or financial relationships that could be construed as a potential conflict of interest.

## References

[B1] Adolfsson R. Gottfries C. G. Roos B. E. Winblad B. (1979). Changes in the brain catecholamines in patients with dementia of Alzheimer type. Br. J. Psychiatry 135, 216–223. 486847 10.1192/bjp.135.3.216

[B2] Allard P. Alafuzoff I. Carlsson A. Eriksson K. Ericson E. (1990). Loss of dopamine uptake sites labeled with [3H]GBR-12935 in Alzheimer's disease. Eur. Neurol. 30, 181–185. 2209670 10.1159/000117341

[B3] Bäckman L. Farde L. (2001). Dopamine and cognitive functioning: Brain imaging findings in Huntington's disease and normal aging. Scand. J. Psychol. 42, 287–296. 10.1111/1467-9450.00238 11501742

[B4] Barbanti P. Fabbrini G. Ricci A. Bruno G. Cerbo R. (2000). Reduced density of dopamine D2-like receptors on peripheral blood lymphocytes in Alzheimer's disease. Mech. Ageing Dev. 120, 65–75. 10.1016/S0047-6374(00)00183-4 11087905

[B5] Barnum D. W. (1977). Spectrophotometric determination of catechol, epinephrine, dopa, dopamine and other aromatic vic-diols. Anal. Chim. Acta 89, 157–166. 10.1016/S0003-2670(01)83081-6 842863

[B6] Belbin O. Morgan K. Medway C. Warden D. Cortina-Borja M.. (2019). The epistasis project: a multi-cohort study of the effects of BDNF, DBH, and SORT1 epistasis on alzheimer's disease risk. J. Alzheimers Dis 68, 1535–1547. 10.3233/JAD-181116 30909233

[B7] Bennett J. P. Burns J. M. (2011). Safety Study of R(+)Pramipexole to Treat Early Alzheimer's Disease. Available online at: https://clinicaltrials.gov/ct2/show/NCT01388478?term=NCT01388478&rank=1 (accessed June 10, 2019).

[B8] Booth T. C. Nathan M. Waldman A. D. Quigley A. M. Schapira A. H.. (2015). The role of functional dopamine-transporter SPECT imaging in parkinsonian syndromes, part 1. AJNR Am. J. Neuroradiol. 36, 229–235. 10.3174/ajnr.A3970 24904053 PMC7965655

[B9] Borba E. M. Duarte J. A. Bristot G. Scotton E. Camozzato A. L.. (2016). Brain-Derived neurotrophic factor serum levels and hippocampal volume in mild cognitive impairment and dementia due to alzheimer disease. Dement. Geriatr. Cogn. Dis. Extra. 6, 559–567. 10.1159/000450601 28101102 PMC5216193

[B10] Casadó-Anguera V. Bonaventura J. Moreno E. Navarro G. Cortes A.. (2016). Evidence for the heterotetrameric structure of the adenosine A2A-dopamine D2 receptor complex. Biochem. Soc. Trans. 44, 595–600. 10.1042/BST20150276 27068975

[B11] Cavić M. Lluís C. Moreno E. Bakešová J. Canela E. I.. (2011). Production of functional recombinant G-protein coupled receptors for heteromerization studies. J. Neurosci. Methods 199, 258–264. 10.1016/j.jneumeth.2011.05.021 21658412

[B12] Cheng L. Doecke J. D. Sharples R. A. Villemagne V. L. Fowler C. J.. (2015). Prognostic serum miRNA biomarkers associated with Alzheimer's disease shows concordance with neuropsychological and neuroimaging assessment. Mol. Psychiatry 20, 1188–1196. 10.1038/mp.2014.127 25349172

[B13] Cortés A. Moreno E. Rodríguez-Ruiz M. Canela E. I. Casadó V. (2016). Targeting the dopamine D3 receptor: an overview of drug design strategies. Expert Opin. Drug Discov. 11, 641–664. 10.1080/17460441.2016.1185413 27135354

[B14] Cortés R. Probst A. Palacios J. M. (1988). Decreased densities of dopamine D1 receptors in the putamen and hippocampus in senile dementia of the Alzheimer type. Brain Res. 475, 164–167. 2975188 10.1016/0006-8993(88)90212-0

[B15] Cross A. J. Crow T. J. Ferrier I. N. Johnson J. A. Markakis D. (1984). Striatal dopamine receptors in Alzheimer-type dementia. Neurosci. Lett. 52, 1–6. 6241300 10.1016/0304-3940(84)90341-0

[B16] Cummings J. C. (2015). Rasagiline Rescue in Alzheimer's Disease Clinical Trial (R2). Available online at: https://clinicaltrials.gov/ct2/show/NCT02359552?term=NCT02359552&rank=1 (accessed June 10, 2019).

[B17] Dayton M. A. Geier G. E. Wightman R. M. (1979). Electrochemical measurement of release of dopamine and 5-hydroxytryptamine from synaptosomes. Life Sci. 24, 917–924. 449600 10.1016/0024-3205(79)90342-4

[B18] De Keyser J. Ebinger G. Vauquelin G. (1990). D1-Dopamine receptor abnormality in frontal cortex points to a functional alteration of cortical cell membranes in Alzheimer's disease. Arch. Neurol. 47, 761–763. 2357156 10.1001/archneur.1990.00530070055011

[B19] Dekker A. D. Vermeiren Y. Carmona-Iragui M. Benejam B. Videla L.. (2018). Monoaminergic impairment in Down syndrome with Alzheimer's disease compared to early-onset Alzheimer's disease. Alzheimers Dement. 10, 99–111. 10.1016/j.dadm.2017.11.001 29780859 PMC5956808

[B20] Devanand D. P. Pelton G. H. Cunqueiro K. Sackeim H. A. Marder K. (2011). A 6-month, randomized, double-blind, placebo-controlled pilot discontinuation trial following response to haloperidol treatment of psychosis and agitation in Alzheimer's disease. Int. J. Geriatr. Psychiatry 26, 937–943. 10.1002/gps.2630 21845596 PMC3685500

[B21] Donthamsetti P. Gallo E. F. Buck D. C. Stahl E. L. Zhu Y. (2018). Arrestin recruitment to dopamine D2 receptor mediates locomotion but not incentive motivation. Mol. Psychiatry. 23, 1580–1595. 10.1038/s41380-018-0212-4 PMC6378141 30120413

[B22] Drayton S. J. Davies K. Steinberg M. Leroi I. Rosenblatt A.. (2004). Amantadine for executive dysfunction syndrome in patients with dementia. Psychosomatics 45, 205–209. 10.1176/appi.psy.45.3.205 15123844

[B23] Ehrin E. Farde L. de Paulis T. Eriksson L. Greitz T.. (1985). Preparation of 11C-labelled Raclopride, a new potent dopamine receptor antagonist: preliminary PET studies of cerebral dopamine receptors in the monkey. Int J Appl Radiat Isot. 36, 269–273. 3874833 10.1016/0020-708x(85)90083-3

[B24] Ferretti M. T. Iulita M. F. Cavedo E. Chiesa P. A. Schumacher D. A.. (2018). Sex differences in Alzheimer disease - the gateway to precision medicine. Nat. Rev. Neurol. 14, 457–469. 10.1038/s41582-018-0032-9 29985474

[B25] Franco R. Casadó V. Cortés A. Ferrada C. Mallol J.. (2007). Basic concepts in G-protein-coupled receptor homo- and heterodimerization. Sci. World J. 7, 48–57. 10.1100/tsw.2007.197 17982576 PMC5901144

[B26] Fribourg M. Moreno J. L. Holloway T. Provasi D. Baki L.. (2011). Decoding the signaling of a GPCR heteromeric complex reveals a unifying mechanism of action of antipsychotic drugs. Cell 147, 1011–1023. 10.1016/j.cell.2011.09.055 22118459 PMC3255795

[B27] Giacobini E. Pepeu G. (2018). Sex and gender differences in the brain cholinergic system and in the response to therapy of alzheimer disease with cholinesterase inhibitors. Curr. Alzheimer Res. 15, 1077–1084. 10.2174/1567205015666180613111504 29895246

[B28] Hagena H. Manahan-Vaughan D. (2016). Dopamine D1/D5, but not D2/D3, receptor dependency of synaptic plasticity at hippocampal mossy fiber synapses that is enabled by patterned afferent stimulation, or spatial learning. Front. Synaptic. Neurosci. 8:31 10.3389/fnsyn.2016.00031 27721791 PMC5033958

[B29] Herrmann N. Rothenburg L. S. Black S. E. Ryan M. Liu B. A.. (2008). Methylphenidate for the treatment of apathy in Alzheimer disease: Prediction of response using dextroamphetamine challenge. J. Clin. Psychopharmacol. 28, 296–301. 10.1097/JCP.0b013e318172b479 18480686

[B30] Higgins J. P. Thompson S. G. Deeks J. J. Altman D. G. (2003). Measuring inconsistency in meta-analyses. BMJ. 327, 557–560. 10.1136/bmj.327.7414.557 12958120 PMC192859

[B31] Jessen F. (2011). Bupropion for the Treatment of Apathy in Alzheimer's Dementia (APA-AD). Available online at: https://clinicaltrials.gov/ct2/show/NCT01047254?term=NCT01047254&rank=1 (accessed June 10, 2019).

[B32] Joyce J. N. Kaeger C. Ryoo H. Goldsmith S. (1993). Dopamine D2 receptors in the hippocampus and amygdala in Alzheimer's disease. Neurosci. Lett. 154, 171–174. 8361636 10.1016/0304-3940(93)90199-u

[B33] Joyce J. N. Murray A. M. Hurtig H. I. Gottlieb G. L. Trojanowski J. Q. (1998). Loss of dopamine D2 receptors in Alzheimer's disease with parkinsonism but not Parkinson's or Alzheimer's disease. Neuropsychopharmacology 19, 472–480. 10.1016/S0893-133X(98)00044-X 9803423

[B34] Karrer T. M. Josef A. K. Mata R. Morris E. D. Samanez-Larkin G. R. (2017). Reduced dopamine receptors and transporters but not synthesis capacity in normal aging adults: a meta-analysis. Neurobiol. Aging 57, 36–46. 10.1016/j.neurobiolaging.2017.05.006 28599217 PMC5645072

[B35] Kemppainen N. Laine M. Laakso M. P. Kaasinen V. Någren K.. (2003). Hippocampal dopamine D2 receptors correlate with memory functions in Alzheimer's disease. Eur. J. Neurosci. 18, 149–154. 10.1046/j.1460-9568.2003.02716.x 12859348

[B36] Koch G. Di Lorenzo F. Bonnì S. Giacobbe V. Bozzali M. (2014). Dopaminergic modulation of cortical plasticity in Alzheimer's disease patients. Neuropsychopharmacology 39, 2654–2661. 10.1038/npp.2014.119 24859851 PMC4207345

[B37] Kumar U. Patel S. C. (2007). Immunohistochemical localization of dopamine receptor subtypes (D1R-D5R) in Alzheimer's disease brain. Brain Res. 1131, 187–196. 10.1016/j.brainres.2006.10.049 17182012

[B38] Kurup R. K. Kurup P. A. (2003). Hypothalamic digoxin, hemispheric chemical dominance, and alzheimer's disease. Int. J. Neurosci. 113, 361–381. 10.1080/00207450390242804 12803139

[B39] Lanctôt K. L. Herrmann N. Black S. E. Ryan M. Rothenburg L. S.. (2008). Apathy associated with Alzheimer disease: use of dextroamphetamine challenge. Am. J. Geriatr. Psychiatry 16, 551–557. 10.1097/JGP.0b013e318170a6d1 18591575

[B40] Li S. C. Lindenberger U. Bäckman L. (2010). Dopaminergic modulation of cognition across the life span. Neurosci. Biobehav. Rev. 34, 625–630. 10.1016/j.neubiorev.2010.02.003 20152855

[B41] Li W. Rossi D. T. Fountain S. T. (2000). Development and validation of a semi-automated method for L-dopa and dopamine in rat plasma using electrospray LC/MS/MS. J. Pharm. Biomed. Anal. 24, 325–333. 10.1016/S0731-7085(00)00422-2 11130211

[B42] Liu L. Li Q. Li N. Ling J. Liu R.. (2011). Simultaneous determination of catecholamines and their metabolites related to Alzheimer's disease in human urine. J. Separ. Sci. 34, 1198–1204. 10.1002/jssc.201000799 21462336

[B43] Lopez-Bastida J. Serrano-Aguilar P. Perestelo-Perez L. Oliva-Moreno J. (2006). Social-economic costs and quality of life of Alzheimer disease in the Canary Islands, Spain. Neurology 67, 2186–2191. 10.1212/01.wnl.0000249311.80411.93 17190942

[B44] Martorana A. Di Lorenzo F. Esposito Z. Lo G. T. Bernardi G. (2013). Dopamine D(2)-agonist rotigotine effects on cortical excitability and central cholinergic transmission in Alzheimer's disease patients. Neuropharmacology 64, 108–113. 10.1016/j.neuropharm.2012.07.015 22863599

[B45] Martorana A. Mori F. Esposito Z. Kusayanagi H. Monteleone F.. (2009). Dopamine modulates cholinergic cortical excitability in Alzheimer's disease patients. Neuropsychopharmacology 34, 2323–2328. 10.1038/npp.2009.60 19516251

[B46] Mattsson N. Andreasson U. Zetterberg H. Blennow K. Weiner M. W.. (2017). Association of plasma neurofilament light with neurodegeneration in patients with Alzheimer disease. JAMA Neurol. 74, 557–566. 10.1001/jamaneurol.2016.6117 28346578 PMC5822204

[B47] Mintzer J. Greenspan A. Caers I. Van Hove I. Kushner S.. (2006). Risperidone in the treatment of psychosis of Alzheimer disease: results from a prospective clinical trial. Am. J. Geriatr. Psychiatry 14, 280–291. 10.1097/01.JGP.0000194643.63245.8c 16505133

[B48] Mitchell R. A. Herrmann N. Lanctôt K. L. (2011). The role of dopamine in symptoms and treatment of apathy in Alzheimer's disease. CNS Neurosci. Ther. 17, 411–427. 10.1111/j.1755-5949.2010.00161.x 20560994 PMC6493833

[B49] Moher D. Liberati A. Tetzlaff J. Altman D. G. (2009). Preferred reporting items for systematic reviews and meta-analyses: the PRISMA statement. BMJ 339:b2535. 10.1371/journal.pmed.1000097 19622551 PMC2714657

[B50] Nam E. Derrick J. S. Lee S. Kang J. Han J. (2018). Regulatory activities of dopamine and its derivatives toward metal-free and metal-induced amyloid-beta aggregation, oxidative stress, and inflammation in alzheimer's disease. ACS Chem. Neurosci. 9, 2655–2666. 10.1021/acschemneuro.8b00122 29782798

[B51] Nardone R. Höller Y. Thomschewski A. Kunz A. B. Lochner P. (2014). Dopamine differently modulates central cholinergic circuits in patients with Alzheimer disease and CADASIL. J. Neural. Transm. 121, 1313–1320. 10.1007/s00702-014-1195-1 24677024

[B52] Navarro G. McCormick P. J. Mallol J. Lluís C. Franco R.. (2013). Detection of receptor heteromers involving dopamine receptors by the sequential BRET-FRET technology. Methods Mol. Biol. 964, 95–105. 10.1007/978-1-62703-251-3_7 23296780 PMC9386282

[B53] Nobili A. Latagliata E. C. Viscomi M. T. Cavallucci V. Cutuli D.. (2017). Dopamine neuronal loss contributes to memory and reward dysfunction in a model of Alzheimer's disease. Nat. Commun. 8:14727. 10.1038/ncomms14727 28367951 PMC5382255

[B54] Nozaki O. Iwaeda T. Kato Y. (1996). Amines for detection of dopamine by generation of hydrogen peroxide and peroxyoxalate chemiluminescence. J. Biol. Chem. 11, 309–313. 9343315 10.1002/(SICI)1099-1271(199611)11:6<309::AID-BIO424>3.0.CO;2-6

[B55] Pan X. Kaminga A. C. Wen S. W. Liu A. (2018a). Catecholamines in post-traumatic stress disorder: a systematic review and meta-analysis. Front. Mol. Neurosci. 11:450. 10.3389/fnmol.2018.00450 30564100 PMC6288600

[B56] Pan X. Wang Z. Wu X. Wen S. W. Liu A. (2018b). Salivary cortisol in post-traumatic stress disorder: A systematic review and meta-analysis. BMC Psychiatry 18:324 10.1186/s12888-018-1910-9 30290789 PMC6173866

[B57] Pohanka M. (2018). Oxidative stress in Alzheimer disease as a target for therapy. Bratisl. Lek. Listy 119, 535–543. 10.4149/BLL_2018_097 30226062

[B58] Roostaei T. Nazeri A. Felsky D. De Jager P. L. Schneider J. A.. (2017). Genome-wide interaction study of brain beta-amyloid burden and cognitive impairment in Alzheimer's disease. Mol. Psychiatry 22, 287–295. 10.1038/mp.2016.35 27021820 PMC5042808

[B59] Saqib M. Bashir S. Li H. Wang S. Jin Y. (2019). Lucigenin-Tris(2-carboxyethyl)phosphine chemiluminescence for selective and sensitive detection of TCEP, superoxide dismutase, Mercury(II), and dopamine. Anal. Chem. 91, 3070–3077. 10.1021/acs.analchem.8b05486 30689357

[B60] Scheltens P. Blennow K. Breteler M. M. de Strooper B. Frisoni G. B.. (2016). Alzheimer's disease. Lancet 388, 505–517. 10.1016/S0140-6736(15)01124-1 26921134

[B61] Seeman P. Bzowej N. H. Guan H. C. Bergeron C. Reynolds G. P. (1987). Human brain D1 and D2 dopamine receptors in schizophrenia, Alzheimer's, Parkinson's, and Huntington's diseases. Neuropsychopharmacology 1, 5–15. 2908095 10.1016/0893-133x(87)90004-2

[B62] Shinohara H. Wang F. (2007). Real-time detection of dopamine released from a nerve model cell by an enzyme-catalyzemd luminescence method and its application to drug assessment. Anal. Sci. 23, 81–84. 10.2116/analsci.23.81 17213629

[B63] Stafanini E. Clement-Cormier Y. (1981). Detection of dopamine receptors in the area postrema. Eur. J. Pharmacol. 74, 257–260. 7327206 10.1016/0014-2999(81)90540-9

[B64] Stang A. (2010). Critical evaluation of the Newcastle-Ottawa scale for the assessment of the quality of nonrandomized studies in meta-analyses. Eur. J. Epidemiol. 25, 603–605. 10.1007/s10654-010-9491-z 20652370

[B65] Sweet R. A. Hamilton R. L. Healy M. T. Wisniewski S. R. Henteleff R.. (2001). Alterations of striatal dopamine receptor binding in Alzheimer disease are associated with Lewy body pathology and antemortem psychosis. Arch. Neurol. 58, 466–472. 10.1001/archneur.58.3.466 11255451

[B66] Tiernan C. T. Ginsberg S. D. He B. Ward S. M. Guillozet-Bongaarts A. L.. (2018). Pretangle pathology within cholinergic nucleus basalis neurons coincides with neurotrophic and neurotransmitter receptor gene dysregulation during the progression of Alzheimer's disease. Neurobiol. Dis. 117, 125–136. 10.1016/j.nbd.2018.05.021 29859871 PMC6278831

[B67] Umegaki H. Ikari H. Nakahata H. Endo H. Suzuki Y.. (2000). Plasma cortisol levels in elderly female subjects with Alzheimer's disease: A cross-sectional and longitudinal study. Brain Res. 881, 241–243. 10.1016/S0006-8993(00)02847-X 11036168

[B68] van Valkenhoef G. Lu G. de Brock B. Hillege H. Ades A. E.. (2012). Automating network meta-analysis. Res. Synth. Methods 3, 285–299. 10.1002/jrsm.1054 26053422

[B69] Wagner J. Palfreyman M. Zraika M. (1979). Determination of dopa, dopamine, dopac, epinephrine, norepinephrine, alpha-monofluoromethyldopa and alpha-difluoromethyldopa in various tissues of mice and rats using reversed-phase ion-pair liquid chromatography with electrochemical detection. J. Chromatogr. 164, 41–54. 541397 10.1016/s0378-4347(00)81570-4

[B70] Westerink B. H. Van Oene J. C. (1980). Evaluation of the effect of drugs on dopamine metabolism in the rat superior cervical ganglion by HPLC with electrochemical detection. Eur. J. Pharmacol. 65, 71–79. 7398778 10.1016/0014-2999(80)90210-1

[B71] White I. R. Barrett J. K. Jackson D. Higgins J. P. (2012). Consistency and inconsistency in network meta-analysis: model estimation using multivariate meta-regression. Res. Synth. Methods 3, 111–125. 10.1002/jrsm.1045 26062085 PMC4433771

[B72] Xiao J. Bergson C. (2013). Detection of cell surface dopamine receptors. Methods Mol. Biol. 964, 3–13. 10.1007/978-1-62703-251-3_1 23296774 PMC4075169

[B73] Yates C. M. Simpson J. Gordon A. (1983). Catecholamines and cholinergic enzymes in pre-senile and senile Alzheimer-type dementia and Down's syndrome. Brain Res. 280, 119–126. 10.1016/0006-8993(83)91179-4 6228286

