# Supplementary material for: Dopamine and Dopamine Receptors in Alzheimer's Disease: A Systematic Review and Network Meta-Analysis
Source: Front Aging Neurosci. 2019 Jul 11;11:175. doi: 10.3389/fnagi.2019.00175 (PMC6637734; doi:10.3389/fnagi.2019.00175)
Supplement: Supplementary file 1 [file Data_Sheet_1.doc]

Search strategies: details of search strategy.

((Alzheimer's Disease[Title/Abstract] OR Alzheimer Syndrome[Title/Abstract] OR Alzheimer Dementia[Title/Abstract] OR Alzheimer[Title/Abstract] OR AD[Title/Abstract])) AND (Dopamine[Title] OR Deoxyepinephrine[Title] OR Dopamine Hydrochloride[Title] OR Hydrochloride, Dopamine[Title] OR Intropin[Title] OR Receptors, Dopamine[Title] OR Dopamine Receptor[Title] OR Receptor, Dopamine[Title] OR Dopamine Receptors[Title] OR Receptors, Dopamine D1[Title] OR Receptors, Dopamine D2[Title] OR Receptors, Dopamine D3[Title] OR Receptors, Dopamine D4[Title] OR Receptors, Dopamine D5[Title])

338 of PubMed

TITLE: (Alzheimer's Disease OR Dementia, Senile OR Dementia OR Alzheimer Syndrome OR Alzheimer Dementia OR Alzheimer OR AD) AND TITLE: (Dopamine OR Deoxyepinephrine OR Dopamine Hydrochloride OR Hydrochloride, Dopamine OR Intropin OR Receptors, Dopamine OR Dopamine Receptor OR Receptor, Dopamine OR Dopamine Receptors OR Receptors, Dopamine D1 OR Receptors, Dopamine D2 OR Receptors, Dopamine D3 OR Receptors, Dopamine D4 OR Receptors, Dopamine D5)

212 of Web of Science

('alzheimer disease':ab OR 'alzheimer syndrome':ab OR 'alzheimer dementia':ab OR 'alzheimer':ab OR 'ad':ab) AND ('dopamine':ti OR 'deoxyepinephrine':ti OR 'dopamine hydrochloride':ti OR 'hydrochloride, dopamine':ti OR 'intropin':ti OR 'receptors, dopamine':ti OR 'dopamine receptor':ti OR 'receptor, dopamine':ti OR 'dopamine receptors':ti OR 'receptors, dopamine d1':ti OR 'receptors, dopamine d2':ti OR 'receptors, dopamine d3':ti OR 'receptors, dopamine d4':ti OR 'receptors, dopamine d5':ti)

458 of Embase

AB ( Alzheimer's Disease OR Alzheimer Syndrome OR Alzheimer Dementia OR Alzheimer OR AD ) AND AB ( Dopamine OR Deoxyepinephrine OR Dopamine Hydrochloride OR Hydrochloride, Dopamine OR Intropin OR Receptors, Dopamine OR Dopamine Receptor OR Receptor, Dopamine OR Dopamine Receptors OR Receptors, Dopamine D1 OR Receptors, Dopamine D2 OR Receptors, Dopamine D3 OR Receptors, Dopamine D4 OR Receptors, Dopamine D5 )

77 of PsycARTICLES
