# Supplementary material for: Dopamine and Dopamine Receptors in Alzheimer's Disease: A Systematic Review and Network Meta-Analysis
Source: Front Aging Neurosci. 2019 Jul 11;11:175. doi: 10.3389/fnagi.2019.00175 (PMC6637734; doi:10.3389/fnagi.2019.00175)
Supplement: Supplementary file 2 [file Table_1.doc]

**Table 1. Rank probabilities for six groups of dopaminergic**

| **Group** | | **Rank 1** | **Rank 2** | **Rank 3** | **Rank 4** | **Rank 5** | **Rank 6** |
| --- | --- | --- | --- | --- | --- | --- | --- |
|  | DA | **0.74** | 0.24 | 0.02 | 0.00 | 0.00 | 0.00 |
| **D1-like** | D1 | 0.00 | 0.00 | 0.01 | 0.07 | 0.28 | **0.64** |
| **D2-like** | D2 | 0.20 | **0.49** | 0.18 | 0.09 | 0.04 | 0.01 |
|  | D3 | 0.01 | 0.14 | **0.46** | 0.28 | 0.10 | 0.01 |
|  | D4 | 0.03 | 0.07 | 0.19 | **0.33** | 0.27 | 0.11 |
| **D2-like** | D5 | 0.03 | 0.06 | 0.13 | 0.23 | **0.31** | 0.24 |
